# Reduced MHC and neutral variation in the Galápagos hawk, an island endemic

**DOI:** 10.1186/1471-2148-11-143

**Published:** 2011-05-25

**Authors:** Jennifer L Bollmer, Joshua M Hull, Holly B Ernest, José H Sarasola, Patricia G Parker

**Affiliations:** 1 Department of Biology, University of Missouri-St. Louis, One University Boulevard, St. Louis, MO 63121, USA; 2 Department of Biological Sciences, University of Wisconsin-Milwaukee, P.O. Box 413, Milwaukee, WI 53201, USA; 3 Wildlife and Ecology Unit, Veterinary Genetics Laboratory, University of California, One Shields Avenue, Davis, CA 95616, USA; 4 Department of Animal Science, University of California, One Shields Avenue, Davis, CA 95616, USA; 5 Department of Population Health and Reproduction, School of Veterinary Medicine, University of California, One Shields Avenue, Davis, CA 95616, USA; 6 Department of Evolutionary Ecology, Estación Biológica de Doñana, Avda. Américo Vespucio, 41092 Sevilla, Spain

## Abstract

**Background:**

Genes at the major histocompatibility complex (MHC) are known for high levels of polymorphism maintained by balancing selection. In small or bottlenecked populations, however, genetic drift may be strong enough to overwhelm the effect of balancing selection, resulting in reduced MHC variability. In this study we investigated MHC evolution in two recently diverged bird species: the endemic Galápagos hawk (*Buteo galapagoensis*), which occurs in small, isolated island populations, and its widespread mainland relative, the Swainson's hawk (*B. swainsoni*).

**Results:**

We amplified at least two MHC class II B gene copies in each species. We recovered only three different sequences from 32 Galápagos hawks, while we amplified 20 unique sequences in 20 Swainson's hawks. Most of the sequences clustered into two groups in a phylogenetic network, with one group likely representing pseudogenes or nonclassical loci. Neutral genetic diversity at 17 microsatellite loci was also reduced in the Galápagos hawk compared to the Swainson's hawk.

**Conclusions:**

The corresponding loss in neutral diversity suggests that the reduced variability present at Galápagos hawk MHC class II B genes compared to the Swainson's hawk is primarily due to a founder event followed by ongoing genetic drift in small populations. However, purifying selection could also explain the low number of MHC alleles present. This lack of variation at genes involved in the adaptive immune response could be cause for concern should novel diseases reach the archipelago.

## Background

Genes at the major histocompatibility complex (MHC) are known for their high levels of polymorphism [1], and for their importance in initiating the adaptive vertebrate immune response by binding to foreign peptides and presenting them to T cells [2]. Class I MHC molecules primarily bind to peptides derived from intracellular pathogens, while class II molecules are associated with extracellular pathogens. MHC variability is maintained through balancing selection, with parasite-mediated natural selection and MHC-dependent sexual selection being the most likely mechanisms [2]. A number of lines of evidence indicate that MHC genes are under selection [3], including an excess of nonsynonymous mutations at the peptide-binding region [4] and the long-term retention of allelic lineages post-speciation (trans-species polymorphism; [5]). Discrepancies between population genetic structure at selectively neutral and MHC loci also provide evidence of selection [6,7], because both neutral and MHC loci are affected by neutral forces (gene flow, genetic drift), but only MHC loci should also be affected by selection.

Many natural populations studied have the high variability expected at MHC loci [8,9]. While population bottlenecks are predicted to result in a loss of variability, balancing selection may counteract the effects of drift unless the effective population size becomes so low relative to the selection coefficient that genes under balancing selection behave in a neutral manner [10,11]. A few studies have found relatively high variability at MHC genes in bottlenecked species with low neutral variability [12-14]. However, most studies of small island [15,16] and mainland [17,18] populations that have undergone severe bottlenecks have documented reduced MHC diversity and concluded that genetic drift had overwhelmed selection (reviewed in [19]).

**Figure 1:**
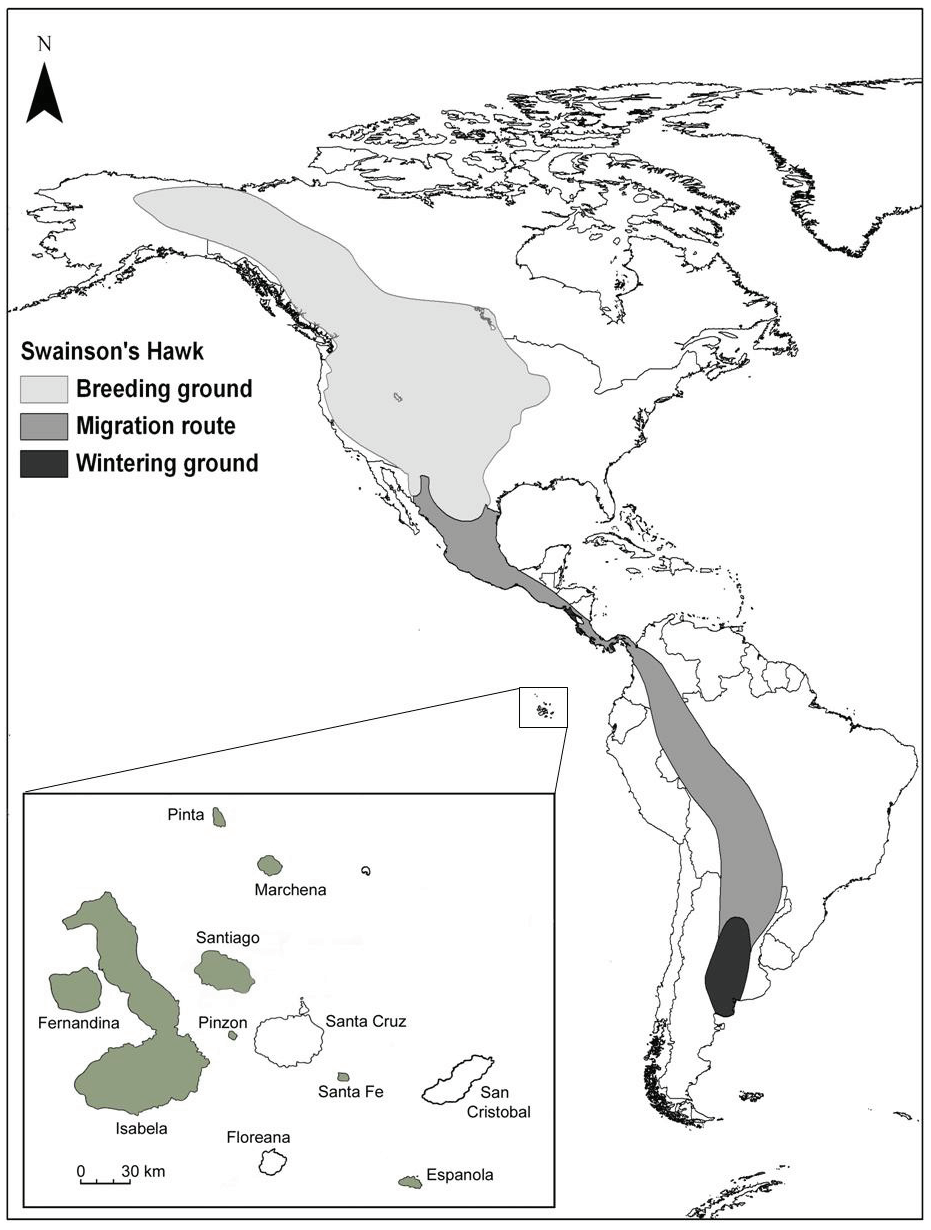
**Distributions of the Galápagos (*Buteo galapagoensis*) and Swainson's hawks (*B. swainsoni*)**. The Galápagos Islands (inset) are located on the equator about 1000 km off the coast of South America. The archipelago is volcanic in origin and there is no evidence that it has ever been connected to the mainland. The Galápagos hawk has breeding populations on all the gray-filled islands; breeding populations have been extirpated from Santa Cruz, San Cristóbal, and Floreana. The Swainson's hawk distribution is from [83]. While the majority of Swainson's hawks overwinter in Argentina, some winter in the southern United States and Mexico.

In this study, we investigated MHC and neutral genetic variation in an island species, the Galápagos hawk (*Buteo galapagoensis*), and its closest mainland relative, the Swainson's hawk (*B. swainsoni*; [20]). Galápagos hawks are endemic to the Galápagos Archipelago and breed on eight islands but were historically distributed on these and three additional islands (Figure 1). No systematic estimate of current population size exists for Galápagos hawks; however, our rough estimates are that two populations consist of several hundred individuals each, while the other six almost certainly are fewer than 100 individuals each.

Previous genetic work revealed low within-population variability and significant between-population differentiation at minisatellite and mitochondrial loci [21,22], indicating little to no current gene flow among islands. In contrast, the migratory Swainson's hawk ([23]; Figure 1), whose population size is unknown but likely numbers at least in the hundreds of thousands based on counts of migrants [24], shows limited population genetic structuring across its western North American breeding range [25]. With their broader distribution and larger population sizes, Swainson's hawks are more variable than Galápagos hawks at minisatellite [26] and mitochondrial loci [22,27]. Mitochondrial data suggest that these two species diverged recently relative to other avian taxa, approximately 125,000 years ago (95% CI: 51,000 - 254,000; [22]). Hull et al. [27] documented mitochondrial paraphyly of the Swainson's hawk relative to the Galápagos hawk, likely a result of incomplete lineage sorting subsequent to colonization.

Here we present the first characterization of MHC class II B genes in Galápagos and Swainson's hawks with the goal of comparing MHC variability in these two species. We predicted that variability would be lower in the Galápagos hawk due to a genetic bottleneck at foundation followed by ongoing genetic drift in these small populations. To better assess the role of genetic drift, we genotyped Galápagos hawks at nuclear microsatellite loci to compare neutral diversity with Swainson's hawks (data from [25]). Finally, we provide a preliminary assessment of MHC evolution in these two closely related species by characterizing the gene copies amplified.

## Results

### MHC diversity

**Figure 2:**
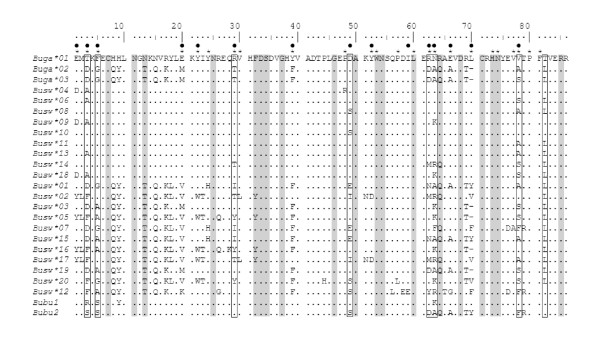
**Alignment of MHC class II B exon 2 amino acid sequences**. Three hawk species are included: *Buteo galapagoensis *(*Buga*), *B. swainsoni *(*Busw*), and *B. buteo *(*Butbu*). The *B. buteo *sequences are from Alcaide et al. [52]. Putative peptide-binding sites based on Brown et al. [28] and Tong et al. [29] are indicated by asterisks and black dots, respectively. Sites identified as conserved by Kaufman et al. [60] are shaded gray, while sites identified by CODEML as being under positive selection by model M8 with a posterior probability >0.99 are in boxes. Periods indicate identity with sequence Buga*01 and dashes indicate deletions.

Sequencing of exon 2 from MHC class II B genes revealed that Galápagos hawks had lower MHC diversity than Swainson's hawks. We recovered three different MHC sequences from 32 Galápagos hawks and 20 sequences from the 20 Swainson's hawks sampled. Each sequence yielded a different amino acid sequence, and no frameshift mutations or stop codons were present (Figure 2). Each Galápagos hawk individual had two or three sequences. One sequence, Buga*01, was present in all individuals. All individuals also had one or both of Buga*02 and Buga*03: twelve individuals had only Buga*02, fifteen had only Buga*03, and five had both. The most parsimonious explanation for this pattern is that the primer set amplified two loci: a locus that is fixed for Buga*01 and a locus that has two alleles, with individuals being homozygous or heterozygous for Buga*02 and Buga*03. Buga*02 and Buga*03 differed by only one base pair; in contrast, Buga*02 and Buga*03 differed from Buga*01 by 30 and 31 bases, respectively, out of 255 bp, and had a one codon deletion not present in Buga*01 (Figure 2). We sampled only four birds per island, so our characterization of the geographic distribution of Buga*02 and Buga*03 is preliminary; however, each was present on at least six of the eight islands: Santa Fe, Pinta, Santiago, and Fernandina had both sequences; Pinzón and Marchena had only Buga*02; and Española and Isabela had only Buga*03. Buga*01 was present on all islands.

From the Swainson's hawks, we recovered 20 different sequences, with each individual having three or four confirmed sequences. Fifth sequences were recovered from three of the individuals; however, we were unable to confirm these because in each case the fifth sequence amplified in only one reaction or did not sequence cleanly. So, every individual had at least two loci. In the 20 birds sampled, we found 18 different MHC genotypes (three birds had the same three sequences). The most common sequence (Busw*08) was recovered from 11 different birds, while 11 of the sequences were recovered from one or two birds. Four of the 20 sequences had a 3 bp deletion at the same codon as the two Galápagos hawk sequences. Of the 255 sites considered, 72 were variable (compared to 31 in Galápagos hawks), and sequences differed by an average of 26.0 ± 12.1 bp.

### Phylogenetic relationships of class II B sequences

**Figure 3:**
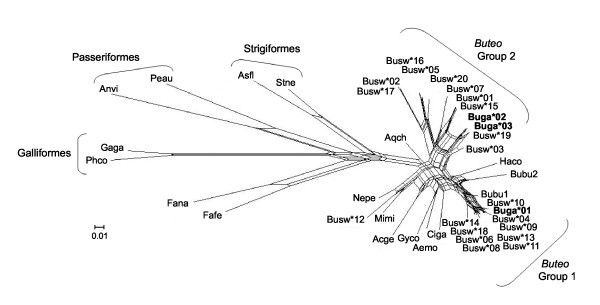
**Phylogenetic network of MHC class II B exon 2 sequences**. Sequences from Galápagos hawks (Buga [in bold], *Buteo galapagoensis*) and Swainson's hawks (Busw, *B. swainsoni*) are included. Also shown are sequences from other members of the order Falconiformes, as well as species from Galliformes, Passeriformes, and Strigiformes (these three orders are labelled, and the rest of the species are from Falconiformes), which we downloaded from GenBank. The network was constructed using the Neighbor-Net method with Jukes-Cantor distances and is based on 255 bp of data. Most of the Galápagos and Swainson's hawk sequences fell into two clusters which are labelled Group 1 and 2. Species and accession numbers of sequences used: Acge, *Accipiter gentilis *[GenBank:EF370917]; Aemo, *Aegypius monachus *[GenBank:EF370890]; Anvi, *Andropadus virens *[GenBank:AY437907]; Asfl, *Asio flammeus *[GenBank:EF641250]; Aqch, *Aquila chrysaetos *[GenBank:EF370905]; Bubu, *Buteo buteo *[GenBank:EF370899-EF370900]; Ciga, *Circaetus gallicus *[GenBank:EF370913]; Haco, *Harpyhaliaetus coronatus *[GenBank:EF370901]; Fafe, *Falco femoralis *[GenBank:EF370951]; Fana, *Falco naumanni *[GenBank:EU107746]; Gaga, *Gallus gallus *[GenBank:AY744363]; Gyco, *Gyps coprotheres *[GenBank:EF370879]; Mimi, *Milvus milvus *[GenBank:EF370897]; Nepe, *Neophron percnopterus *[GenBank:EF370893]; Phco, *Phasianus colchicus *[GenBank:AJ224352]; Peau, *Petroica australis australis *[GenBank:AY428567]; Stne, *Strix nebulosa *[GenBank:EF641241]

A phylogenetic network revealed structuring among Galápagos and Swainson's hawk sequences (Figure 3). Galápagos and Swainson's hawk sequences were more similar to other sequences from Falconiformes than to those from other orders. Nine Swainson's hawk sequences and the fixed Galápagos hawk sequence (Buga*01) formed a cluster designated Group 1, ten Swainson's and the two remaining Galápagos hawk sequences formed a second group (Group 2), and one Swainson's sequence (Busw*12) was divergent from the rest, being more similar to sequences from other species. All six sequences with the codon deletion were in Group 2. One common buzzard (*Buteo buteo*) sequence (*Bubu1*) also fell into Group 1, while the second buzzard sequence (*Bubu2*) had characteristics of both groups; the first two-thirds of the sequence closely matched Group 1, while the remainder matched Group 2 (Figure 2). Sequences within Group 1 were less divergent than those within Group 2 (Figures 2, 3; Table 1). The two groups had similar numbers of sequences (10 in Group 1 and 12 in Group 2); however, sequences in Group 2 had 53 variable sites and differed by an average of 22.3 ± 9.1 bases, whereas sequences in Group 1 had only 16 variable sites and differed by an average of 5.9 ± 3.0 bases. Group 1 and Group 2 sequences differed by an average of 32.5 ± 4.6 bases. Busw*12, present in only one individual, was more divergent than the other sequences. It differed from Group 1 sequences by an average of 39.1 ± 2.0 bases out of 255 and from Group 2 by an average of 36.4 ± 5.4 bases. Galápagos and Swainson's hawks did not share any sequences, and within-group diversity was lower in the Galápagos hawk than in the Swainson's hawk (Table 1).

**Table 1:** Sequence diversity within Galápagos (*Buteo galapagoensis*) and Swainson's (*B. swainsoni*) hawks at MHC class II B loci

Species	*N*	No. sequences	No. polymorphic sites	*π*
*B. galapagoensis *(Grp 1)	32	1	n/a	n/a
*B. galapagoensis *(Grp 2)	32	2	1	0.004
*B. swainsoni *(Grp 1)	20	9	16	0.023
*B. swainsoni *(Grp 2)	20	10	53	0.090

The sample size of individuals sequenced (*N*), number of different sequences recovered, number of polymorphic sites, and nucleotide diversity (*π*) are given. The statistics are based on 255 bp of exon 2.

### Positive selection on exon 2

We found evidence for positive selection on putative peptide-binding codons (PBCs), with results being very similar for PBCs identified by Brown et al. [28] and Tong et al. [29] (Table 2). Analyzing all of the sequences together, rates of nonsynonymous substitutions were significantly greater than synonymous substitutions at PBCs but not at the remaining codons. The same was true when Swainson's hawks were analyzed separately; we did not analyze Galápagos hawks separately due to the paucity of sequences. Substitution rates were higher in Group 2 than Group 1; 93.3% of PBCs (based on Tong et al. [29]) were polymorphic in Group 2 and only 46.7% in Group 1. We also found evidence of positive selection using the maximum likelihood method implemented in CODEML. Both selection models (M2a, M8) provided a better fit than their respective neutral models (M1a, M7) for all three sequence sets tested (P < 0.001; Table 3). The M8 models identified a total of eight sites as being under positive selection with posterior probabilities >0.99 (Table 3). The sites varied between Group 1 and 2 sequences: two were significant in Group 1 only, three in Group 2 only, and three in both. Of the eight sites identified by CODEML as being under positive selection, six were designated by Brown et al. [28] and seven by Tong et al. [29] as being peptide-binding (Figure 2).

**Table 2:** Comparison of non-synonymous (*d*_N_) and synonymous (*d*_S_) substitution rates at putative peptide-binding codons (PBCs) and non-PBCs

			PBCs			Non-PBCs		
Method	Sequence set	No. of sequences	*d*_N _± SE	*d*_S _± SE	*d*_N_/*d*_S_	*d*_N _± SE	*d*_S _± SE	*d*_N_/*d*_S_
Brown et al. 1993	All	23	0.272 ± 0.073	0.031 ± 0.019	8.77***	0.069 ± 0.017	0.109 ± 0.037	2.95
	*B. swainsoni*	20	0.268 ± 0.076	0.027 ± 0.017	9.82***	0.073 ± 0.017	0.114 ± 0.042	0.64
	Group 1	10	0.072 ± 0.029	0.007 ± 0.007	10.43*	0.014 ± 0.009	0.010 ± 0.010	1.40
	Group 2	12	0.299 ± 0.085	0.036 ± 0.025	8.25***	0.045 ± 0.014	0.092 ± 0.034	0.49
Tong et al. 2006	All	23	0.573 ± 0.127	0.060 ± 0.034	9.55***	0.057 ± 0.013	0.090 ± 0.030	0.63
	*B. swainsoni*	20	0.566 ± 0.119	0.054 ± 0.033	10.48***	0.060 ± 0.014	0.094 ± 0.032	0.64
	Group 1	10	0.123 ± 0.045	0.011 ± 0.011	11.18**	0.009 ± 0.007	0.010 ± 0.011	0.90
	Group 2	12	0.607 ± 0.150	0.074 ± 0.044	8.20***	0.036 ± 0.010	0.080 ± 0.029	0.45

Separate analyses were run using all sequences, *Buteo swainsoni *sequences, Group 1, and Group 2. Codons were designated as peptide-binding or non-peptide-binding using two methods: Brown et al. [28] and Tong et al. [29]. Significance levels of Z-tests for positive selection (H_A_: *d*_N _>*d*_S_) are indicated by asterisks (P < 0.05*, P < 0.01**, P < 0.001***).

**Table 3:** Evidence of positive selection on Galápagos and Swainson's hawk MHC class II B exon 2 sequences

Sequence set	Model	ln *L*	Parameter estimates	Positively selected sites	LRT statistic
All sequences	M1a	-1151.58	p = 0.775 (p_1 _= 0.225), ω = 0.071, ω_1 _= 1	not allowed	
	M2a	-1106.64	p_0 _= 0.559, p_1 _= 0.379 (p_2 _= 0.062), ω_0 _= 0.078, ω_1 _= 1, ω_2 _= 9.80	3, 5, 29, 49, 62, 63, 78	89.9 (P < 0.001)
	M7	-1157.11	p = 0.145, q = 0.334	not allowed	
	M8	-1106.97	p_0 _= 0.938 (p_1 _= 0.062) p = 0.104, q = 0.117, ω = 10.11	3, 5, 29, 49, 62, 63, 78, 82	100.3 (P < 0.001)
Group 1	M1a	-482.72	p = 0.770 (p_1 _= 0.230), ω = 0.0, ω_1 _= 1	not allowed	
	M2a	-465.93	p_0 _= 0.884, p_1 _= 0.00 (p_2 _= 0.116), ω_0 _= 0.00, ω_1 _= 1, ω_2 _= 48.82	49, 63, 78, 82	33.6 (P < 0.001)
	M7	-482.79	p = 0.005, q = 0.020	not allowed	
	M8	-465.93	p_0 _= 0.884 (p_1 _= 0.116) p = 0.005, q = 16.87, ω = 48.82	3, 49, 63, 78, 82	33.7 (P < 0.001)
Group 2	M1a	-825.63	p = 0.683 (p_1 _= 0.317), ω = 0.054, ω_1 _= 1	not allowed	
	M2a	-793.03	p_0 _= 0.491, p_1 _= 0.432 (p_2 _= 0.077), ω_0 _= 0.00, ω_1 _= 1, ω_2 _= 16.66	5, 29, 63, 78	65.2 (P < 0.001)
	M7	-828.09	p = 0.096, q = 0.149	not allowed	
	M8	-793.04	p_0 _= 0.922 (p_1 _= 0.078) p = 0.023, q = 0.028, ω = 16.13	5, 29, 49, 62, 63, 78	70.1 (P < 0.001)

Separate analyses were performed on all sequences (N = 23), Group 1 (N = 10), and Group 2 (N = 12). Log-likelihood values and parameter estimates calculated by CODEML are presented, as are the sites predicted by models M2a and M8 to be under positive selection with posterior probabilities >0.99 and likelihood ratio test (LRT) statistics. LRTs were used to compare M2a to M1a and M8 to M7. The test statistics were compared to a chi-square distribution with df = 2. The positively selected sites are numbered according to the alignment in Figure 2.

### Neutral variability

Galápagos hawks have low diversity at microsatellite loci as well. We found a total of 78 alleles across 17 loci in the 185 individuals genotyped. For the seven populations, mean allelic richness varied between 1.53 and 3.29. This low variability does not appear to be the result of a recent bottleneck. The program Bottleneck reported a significant excess of heterozygosity in only one (Pinta; *P *= 0.027) of the seven populations; however, sample sizes may have been too small to provide statistical resolution.

Microsatellite variation was lower in Galápagos hawks than in Swainson's hawks. For a more direct comparison, we pooled the Galápagos hawk populations and re-evaluated them using only the 13 loci that had also been used to genotype Swainson's hawks [25]: BswA110w, BswA204w, BswA302w, BswA317w, BswB220w, BswD107w, BswD122w, BswD127w, BswD210w, BswD220w, BswD310w, BswD313w, and BswD324w. Galápagos hawks had significantly lower mean allelic richness, expected heterozygosity and observed heterozygosity than Swainson's hawks (Wilcoxon signed rank tests: Z = -3.18, p < 0.001; Table 4).

**Table 4:** Population genetic parameters for Galápagos and Swainson's hawk populations estimated from microsatellite data

Species	No. of loci	Population	*N*	*A*_R _± SD	Gene diversity ± SD	*H*_o _± SD
Galápagos hawk	17	Santa Fe	17	1.76 ± 1.20	0.13 ± 0.21	0.13 ± 0.23
		Española	17	1.53 ± 0.72	0.14 ± 0.23	0.13 ± 0.20
		Pinta	26	1.93 ± 1.17	0.24 ± 0.28	0.23 ± 0.26
		Marchena	22	1.84 ± 1.13	0.18 ± 0.25	0.20 ± 0.31
		Fernandina	24	2.65 ± 1.77	0.32 ± 0.31	0.31 ± 0.31
		Santiago	54	3.29 ± 1.86	0.41 ± 0.29	0.40 ± 0.30
		Isabela	25	3.40 ± 2.21	0.41 ± 0.30	0.39 ± 0.30
Galápagos hawk	13	All pops	185	4.92 ± 2.78	0.50 ± 0.29	0.34 ± 0.24
Swainson's hawk	13	Western U.S.	301	19.29 ± 9.75	0.87 ± 0.06	0.87 ± 0.06

Allelic richness (*A*_R_), gene diversity, and observed heterozygosity (*H*_o_) are given. For the Galápagos hawk, we present measures from each population individually using all 17 polymorphic loci, and then we present measures for all populations combined at the 13 loci that were also used to genotype Swainson's hawks [34].

## Discussion

While MHC peptide-binding genes typically display high variability, in some cases small or bottlenecked populations are reported to exhibit reduced variation. We had predicted that MHC class II B variability would be lower in the endemic Galápagos hawk than in the mainland Swainson's hawk due to a colonization event followed by ongoing genetic drift in the small island populations. We found that Galápagos hawks had fewer, less divergent sequences than Swainson's hawks. A corresponding low level of neutral microsatellite variability suggests that drift has played a strong role in shaping MHC variation in Galápagos hawks.

### Low genetic diversity in the Galápagos hawk

Galápagos hawks exhibited low MHC class II B diversity, with all 32 individuals having Buga*01 (possibly a fixed locus) and one or both of Buga*02 and Buga*03 (possibly a second locus). Fixed loci have been reported in other species, including an island rat [30] and bottlenecked populations of the Eurasian beaver [18]; however, in those cases populations were fixed for different alleles. Likewise, other island populations have fewer MHC alleles compared to a mainland relative [9,30,31]. The reduced set of alleles found in the island populations of Eurasian kestrel [9] and Seychelles warbler [31] were just as divergent as alleles present in mainland populations. In contrast, Buga*02 and Buga*03 differ by only one base. Exon 2 alleles typically differ by a larger number of bases; for example, lesser kestrel sequences differ by an average of 22.7 bases [9]. So, it is more likely that one of these Galápagos hawk alleles arose from the other through point mutation, than both being retained ancestral alleles. Similarly, the endangered Galápagos penguin has only three sequences (differing by 1-3 bp) at one locus, suggesting the penguins were once fixed for a single allele also [16]. Interestingly, none of the Galápagos sequences was present among the Swainson's hawks sampled, which could be because those sequences were rare in the ancestral population or they mutated from ancestral sequences after colonization.

The low genetic diversity present at neutral markers provides strong evidence for the role of a founder event and ongoing genetic drift within the Galápagos hawk. Allelic richness and heterozygosity at microsatellite loci were lower in Galápagos populations than in the Swainson's hawk population. A similar pattern of low diversity occurs at minisatellite loci; individuals within populations share an average of 69-96% of their minisatellite alleles [21], while an average of 20-30% is more typical for outbred populations [32]. At the mitochondrial control region, Galápagos hawks had five haplotypes that were on average less divergent than the 36 haplotypes in Swainson's hawks [27], and seven of eight populations appear fixed for single haplotypes at almost 3 kb of mitochondrial sequence [22].

The pattern of MHC variation in the Galápagos hawk is likely the result of a loss of ancestral variability at the time of colonization. The apparent fixation of Buga*01 and possible past fixation of Buga*02/03 is more consistent with an extreme bottleneck than ongoing drift. Also, Buga*01 is present on all eight islands; the other two sequences are each present on at least six islands, with at least four populations having both. Minisatellite and mitochondrial data indicate little current gene flow among populations [21,22], so the geographic distribution of the sequences suggests that MHC variability was reduced at or soon after foundation and that the hawks carried the reduced set of alleles with them as they colonized the various islands. Minisatellite data also hint at an early reduction in genetic variability because of high background similarity across all populations [21], and four of the populations (Pinta, Marchena, Santiago, Santa Fe) are fixed for the same mitochondrial haplotype [22].

In addition to drift, low MHC variability in the Galápagos hawk could be attributed to relaxed selection or purifying selection. Parasite diversity on islands may be lower than on the mainland [33], so island populations may experience reduced selection pressure, resulting in less MHC variation being maintained [34]. Island kestrels that experienced lower pathogen diversity and prevalence than mainland kestrels also had lower MHC variability [9]. Galápagos hawks harbour five ectoparasite species and an undescribed *Trypanosoma *species [26,35], but Swainson's hawks are likely exposed to a greater diversity of both endo- and ectoparasites. For example, Swainson's hawks carry five louse species [36], while Galápagos hawks carry three. Swainson's hawks are migratory and should encounter different sets of pathogens at their breeding and wintering grounds, which has been hypothesized to lead to greater selection for variability at the MHC [6]. Alternatively, the lack of MHC variation could be explained by purifying selection for alleles advantageous against a current parasite or a past selective sweep [37]. These alternatives do not explain the corresponding low variability at neutral markers, but they cannot be ruled out with our dataset and could be occurring in addition to drift.

While studies have demonstrated a relationship between MHC diversity and resistance to parasites [38,39], the consequences of low MHC diversity remain unclear. Low MHC diversity has been implicated in the rapid spread of an infectious cancer that has caused declines in Tasmanian devil populations [40]. However, other species appear to have experienced little negative impact, with some able to undergo population expansions [18,41] and survive thousands of years [42]. Radwan et al. [19] concluded that most bottlenecked populations do lose MHC variation, but data demonstrating an associated population decline or extinction are scarce, although they point out that studies are biased toward populations that have survived past bottlenecks. In some bottlenecked populations, the remaining alleles are divergent [17,43], and it is possible that this variation is sufficient for survival under current environmental conditions. The introduction of novel diseases may pose the greatest threat, as genetically uniform species may be less capable of adapting. Whiteman et al. [26] found that smaller, more inbred (as measured at minisatellites) Galápagos hawk populations had higher loads of a coevolved body louse and, in general, lower and less variable natural antibody titres than the larger populations. This suggests that genetic variability may indeed affect this species' ability to mount an effective immune response.

### Characterization of MHC genes

Class II B genes are prone to duplication and deletion events [44], and gene number may vary both within and between species [45,46]. Among birds, it appears that two class II B genes were present before the major avian radiations [47], and existing bird taxa range in gene copy number from one or two [48,49] to seven or more [50,51]. The number of sequences we recovered from each hawk (≤ 4) suggests we amplified two loci, which is similar to the one to two loci amplified from other accipitrid species [52]. However, we cannot be certain, so two loci is a minimum estimate; it is possible that the primer set we used did not amplify all exon 2 sequences or genes actually present. A more thorough investigation of the class II architecture of these species is needed to determine the true number of genes.

Assignment of alleles to particular MHC class II B genes based on exon 2 has proven difficult in birds, possibly because recent gene duplication or elevated rates of gene conversion have resulted in higher intergenic similarity [53,54]. However, the hawk sequences exhibited substructuring, clustering into two groups that may represent separate genes. Group 1 was notable because of its low sequence divergence compared to sequences in Group 2. Other studies of avian MHC have also identified genes or clusters of sequences with low divergences, mostly in passerines [55] but also the Y complex in Galliformes [56]. Some low variability genes appear to be nonfunctional pseudogenes, having mutations that prevent transcription [57,58], while others are nonclassical with limited expression and specialized functions [56,59].

Because we used genomic DNA, we cannot be certain that the hawk sequences we amplified are expressed. No frameshift mutations or stop codons were present within the region sequenced, and evolutionarily conserved amino acid residues occurred at 17 of the 19 sites thought to be functionally important for class II molecules (Figure 2; [60]). Also, an excess of nonsynonymous substitutions was present in both groups of sequences, which is evidence that selection has acted on these loci, although not necessarily recently [3], and Group 2 sequences had genetic distances similar to those of expressed sequences from classical MHC loci in other species. More sequence data and expression analyses are needed to better characterize these genes and to determine if the Group 1 sequences are from pseudogenes or specialized genes. The presence of a Group 1 sequence in the Old World common buzzard suggests that allelic lineage predates the divergences of these *Buteo *species.

## Conclusions

Here, we documented low MHC variability in an island endemic, the Galápagos hawk, compared to its closest mainland relative, the Swainson's hawk. The corresponding loss of genetic diversity at neutral markers (microsatellite, minisatellite, and mitochondrial loci) suggests that a founder event at colonization followed by ongoing drift in small populations is the primary cause of low MHC diversity. However, purifying selection or a past selective sweep could also explain the low number of MHC alleles present. The Galápagos hawk's low genetic variability may affect its ability to mount an immune response [26] and could be cause for concern should novel diseases reach the archipelago.

## Methods

### Sampling

Galápagos hawks (n = 189) were sampled from 1998 to 2003 on eight islands encompassing the entire breeding range of the species: Española, Santa Fe, Pinzón, Santiago, Isabela, Fernandina, Marchena, and Pinta. Overwintering Swainson's hawks (n = 20) were sampled in 2003 at a communal roost near Las Varillas, Córdoba province, Argentina. Both radio-tracking [23] and stable isotope [61] data show that Swainson's hawks from different breeding populations intermix on the Argentine wintering grounds. Therefore, it is likely that our sample is derived from more than one breeding population; however, our measure of Swainson's hawk variability is still an underestimate of the variability present in the species. We banded each hawk and took morphological measurements as well as two 50 μl blood samples for genetic analyses (see Bollmer et al. [21] and Whiteman et al. [26] for more details).

### MHC genotyping

At the MHC, we genotyped four Galápagos hawks at class II B genes from each of the eight island populations (using unrelated adults from different territories) for a total of 32 individuals. With this sampling, we intended to gauge overall variability at the species level rather than evaluate the amount of variability within individual populations. The twenty Las Varillas Swainson's hawks were also genotyped at the MHC. Individuals that had been used in previous population genetic studies were preferentially chosen [21,22]. We targeted exon 2, which codes for the peptide-binding region of the class II B molecule and has been shown to be under balancing selection [4]. We first amplified a 307 bp fragment (primers included) using the primers Acc2FC and Acc2RC developed by Alcaide et al. [52] from other diurnal raptors. Acc2FC begins in intron 1 and extends 9 bp into exon 2, whereas Acc2RC comprises bases 9 through 27 of intron 2. This PCR amplification was carried out in 40 μl reactions using 1.25X buffer, 0.25 mM dNTPs, 2.5 mM MgCl_2_, 0.5 μM of each primer, 1 U Bioline *Taq *DNA polymerase, and 100 ng of genomic DNA. Reaction conditions were as follows: 94°C for 4 min, then 35 cycles of 94°C for 40 sec, 56°C for 40 sec, and 72°C for 1 min, and a final extension of 72°C for 5 min. We used QIAquick gel extraction kits (QIAGEN, Valencia, CA) to gel-purify the PCR products and then cloned them using the pGEM-T easy vector cloning kit (Promega, Madison, WI). Positive clones were sequenced on an ABI 3100 sequencer using BigDye chemistry (Life Technologies, Carlsbad, CA). Using sequences aligned from Galápagos and Swainson's hawks, we developed a new reverse primer ButeoR (5'-TTC TGG CAC RCA CTC ACC TC-3'), which overlaps the final 3 bp of exon 2 and extends into intron 2.

We employed denaturing gradient gel electrophoresis (DGGE) to genotype the 52 individuals. We screened eight of these individuals using cloning as well and confirmed that genotypes from DGGE and cloning were consistent. We amplified a 298 bp fragment (primers included) using the primers Acc2FC and ButeoR with a GC-clamp applied to the 5' end of ButeoR to facilitate separation of alleles on gels [62]. Reaction conditions were the same as above, and PCR products were run on 8% 19:1 acrylamide/bisacrylamide gels using a 25 to 35% denaturing gradient of formamide and urea. We ran gels for 4.5 h at 160 V at a constant temperature of 60°C, stained them with SYBR^© ^gold (Promega) and then visualized them on a Kodak IS440CF imaging system. In order to obtain the sequences of the alleles, we cut bands out of the gels, suspended them in 50 μl of dH_2_O, re-amplified them using the Acc2FC/ButeoR primer set, and then sequenced them using those same primers. All DGGE bands were cut out and sequenced at least once. Because spurious alleles may form when amplifying multiple sequences in one reaction [63], we considered sequences to be confirmed only if they were amplified in at least two independent reactions. Confirmed sequences are available online [GenBank:EU876805 - EU876827].

### Microsatellite genotyping

We genotyped 185 individual Galápagos hawks at 22 microsatellite loci (BswA110w, BswA204w, BswA302w, BswA303w, BswA312w, BswA317w, BswB111a2w, BswB220w, BswD107w, BswD122w, BswD123w, BswD127w, BswD210w, BswD220w, BswD223w, BswD234w, BswD235w, BswD310w, BswD312w, BswD313w, BswD324w, BswD330w) using the protocol described in Hull et al. [64]. A subset of these loci have been previously used in an examination of Swainson's hawk populations [25]. PCR fragments were size-separated on a 3730 DNA Analyzer (Applied Biosystems, Inc.), and alleles were scored with STRand version 2.3.89 [65]. The 185 individuals represented seven of the eight island populations; our sample size from Pinzón was too small to include.

### Data analysis

For the MHC data, we assembled and edited sequences using SeqMan Pro v. 7.1 (DNASTAR, Inc.) and aligned them in BioEdit [66]. We recovered three or more sequences from most individuals, indicating that two or more gene duplications were amplified. This is consistent with previous data from other accipitrid species where one or two genes were recovered [52]. Co-amplification of multiple genes is common in studies of avian MHC, as the high similarity of duplicated genes often makes it difficult to amplify them individually and to assign sequences to particular genes [8].

We calculated MHC sequence diversity measures using 255 bp of exon 2 (the bases within the primer region were excluded) both within and between species in the program DnaSP v. 4.50.3 [67]. To evaluate relationships among sequences, we constructed a phylogenetic network using the program SplitsTree4 [68]. We employed the Neighbor-Net method [69] using Jukes-Cantor distances. As opposed to traditional phylogenetic trees, phylogenetic networks permit visualization of conflicting signals from processes such as gene duplication and recombination [69]. We tested for evidence of balancing selection on the peptide-binding region by calculating nonsynonymous (*d*_N_) and synonymous (*d*_S_) substitution rates. A *d*_N_/*d*_S _ratio of ω = 1 is expected under neutral evolution, ω < 1 under purifying selection, and ω > 1 under positive selection. First, we calculated the substitution rates using the Nei and Gojobori [70] method with the Jukes-Cantor correction in MEGA v. 4 [71]. Rates were calculated separately for both putative peptide-binding and non-peptide-binding codons as assigned by Brown et al. [28] and Tong et al. [29] for human class II molecules, and Z-tests were used in MEGA to test for positive selection. We also tested for positive selection using the maximum likelihood method implemented in CODEML in the package PAML v. 4 [72,73]. With this method we did not need to make *a priori *assumptions about which codons may be peptide-binding. We used a likelihood ratio test to compare model M1a, a neutral model with two site classes (ω_0 _< 1, ω_1 _= 1), and M2a which incorporates a third site class (ω_2 _> 1) allowing for positive selection [74]. Similarly, we compared M7, a null model with a beta distribution (0 < ω < 1), and M8, which uses a beta distribution but also allows for positive selection [72]. The models were compared using likelihood ratio tests. The test statistics were calculated as two times the difference between the likelihoods of the two models, and they were compared to the Chi-square distribution with degrees of freedom equal to the difference in the number of parameters for the two models (M1a and M7 each have 2 parameters; M2a and M8 have 4). M7 and M8 are the most robust to the effect of recombination, which may cause false positives [75]. Positively selected codons with a ω > 1 were identified using the Bayes empirical Bayes approach [73].

For the microsatellite data, we tested for Hardy-Weinberg equilibrium by locus and population using a randomization test that employs the F_IS _statistic in FSTAT version 2.9.3 [76]. We tested for linkage disequilibrium between all pairs of loci within each population via randomization tests employing the log-likelihood ratio G-statistic in FSTAT. We tested for the presence of null alleles in MICROCHECKER [77]. Bonferroni tests were used to correct for multiple comparisons. Of the 22 loci, three had significant departures from Hardy-Weinberg equilibrium in at least one population (P < 0.0003, the adjusted critical value): BswA303w in Pinta, BswA312w in Fernandina, Isabela, and Pinta, and BswD234w in Española and Pinta. We found evidence of linkage for three pairs of loci (P < 0.00022, the adjusted critical value): BswD312w x BswD235w, BswA303w x BswD234w, and BswD123w x BswD223w. To eliminate Hardy-Weinberg and linkage issues, we removed BswA303w, BswA312w, BswD234w, BswD235w, and BswD223w from further analyses and used the remaining 17 loci. We found no evidence of null alleles among these 17 loci.

We calculated microsatellite allelic richness as the number of alleles per locus after controlling for differences in sample size using rarefaction analysis [78,79] in FSTAT. Average gene diversity and observed heterozygosity were calculated using Arlequin v. 3.1 [80]. We tested for evidence of a recent bottleneck (a significant excess of heterozygosity) in each of the Galápagos hawk island populations using the program BOTTLENECK [81,82]. We used Wilcoxon signed-rank tests under the two-phase model (TPM) of microsatellite evolution with the stepwise mutation model (SMM) set at 70% and the infinite alleles model (IAM) at 30%. We checked the sensitivity of the data to the mutational model by running additional trials using multiple SMM/IAM combinations.

## Authors' contributions

JB developed the project, participated in Galápagos hawk sample collection, did the MHC laboratory work and data analysis, and wrote the first draft of the manuscript. JH performed the microsatellite laboratory work and data analysis, in close consultation with HE. JS collected the Swainson's hawk samples. PP was the primary supervisor and participated in the study's design and coordination. All authors were involved in the writing, and read and approved the final manuscript.
